# Hyperbaric oxygen therapy for the treatment of Steinberg I and II avascular necrosis of the femoral head: a report of fifteen cases and literature review

**DOI:** 10.1007/s00264-021-05120-3

**Published:** 2021-07-04

**Authors:** Motasem Salameh, Isam S. Moghamis, Osama Kokash, Ghalib O. Ahmed

**Affiliations:** 1 Orthopedics Department, Hamad General Hospital, Hamad Medical Corporation, PO Box 3050, Doha, Qatar; 2 HBOT Department, Hamad General Hospital, Hamad Medical Corporation, PO Box 3050, Doha, Qatar; 3 Weil Cornell Medical College, Al Rayyan, Qatar

**Keywords:** Avascular necrosis, Hyperbaric oxygen, Outcome, Pain

## Abstract

**Purpose:**

This article aimed to report a case series of pre-collapse avascular necrosis of the femoral head treated with hyperbaric oxygen and review the most recent literature on the topic.

**Methods:**

The data from a prospectively followed registry of 15 patients with Steinberg I and II avascular necrosis of the femoral head was collected. Functional outcome, pain scores, and radiographic changes at an average follow-up of 22 months were analyzed and reported.

**Results:**

Thirteen patients had satisfactory outcome at final follow-up with an average Oxford hip score of 37.3, pain scores were significantly improved at final follow-up (P < 0.001), and 26.7% of hips progressed to collapse on follow-up radiographs with no complications reported in all patients.

**Conclusion:**

Hyperbaric oxygen treatment for pre-collapse avascular necrosis of the femoral head is considered a safe alternative with satisfactory clinical and radiological outcomes and low complications rate.

## Introduction

Avascular necrosis (AVN) of the femoral head is not an uncommon pathology with debilitating effects on both patients and health systems. Early diagnosis and intervention can lift the burden of major surgical intervention such as joint arthroplasty or arthrodesis [1]. Pre-collapse lesions are typically treated with either medical (bisphosphonates) or surgical (core decompression) modalities with good results [2–5].

Hyperbaric oxygen (HBO) was introduced as a less invasive alternative to surgical interventions for pre-collapse lesions. It is established that HBO improves microcirculation and decreases bone edema, thus helping in pain relief and preventing further head collapse [6]. This article reports a cohort of 15 patients treated with HBO for AVN of the femoral head plus a literature review on the use of HBO for pre-collapse femoral head necrosis.

## Materials and methods

After institutional review board approval, the data was collected from a prospectively followed registry of patients between January 2010 and December 2018. The patient’s demographics (age, gender, BMI), comorbidities, risk factors for AVN (diabetes mellitus, hypertension, alcohol intake, smoking, steroids use, sickle cell disease, chemo or radiation therapy), pre- and post-treatment radiological stage (Steinberg’s classification), functional outcome (Oxford hip score, SF 12), pain score, complications, and the need for further surgical intervention or total hip arthroplasty (THA) were collected. The inclusion criteria for participation in the study were adult patients 18 years and older, with non-traumatic Steinberg [7] stage I–II AVN of the femoral head that was confirmed on MRI and those who underwent hyperbaric oxygen therapy with a minimum follow-up of one year were included. Patients with traumatic AVN, other stages of AVN (stages III, IV, V, and VI), and less than one year follow-up were excluded. Written informed consent was obtained from all patients and they were interviewed through their regular clinical follow-up.

### Clinical and radiological evaluation

The clinical outcome was measured using the Oxford hip score (OHS) and the 12-item short form survey (SF12) for both groups; the forms were used in two languages, namely Arabic and English, and the Arabic form was translated through the institutional medical research centre by a certified translator. The visual analog scale (VAS) was used to assess the patients’ degree of pain pre-treatment and at last follow-up. The radiological progression of the AVN stage was assessed by comparing radiographs before and after treatment. All radiographs and MRI images were reviewed and staged by a musculoskeletal radiologist. Satisfactory clinical outcome was defined as an OHS score of over 30 and no further surgical interventions needed. Radiological progression was identified with more advanced stages in post-treatment radiographs.

After optimization of patients, each patient received between 25 and 40 sessions of HBO therapy, with three to four sessions per week to avoid any possibility of oxygen toxicity. The treatment protocol was delivered by an HBO therapy specialist and involved breathing 100% oxygen at 22.5 lb per square inch (2.2 atm) in a hyperbaric oxygen pressure chamber for 90 min, with additional 15 min for decompression until reaching 22.5 lb per square inch, and two air breaks of five minutes and 15 minutes for recompression back to surface. Each patient was provided with a well-sealed breathing mask from which he or she received the oxygen treatment. Clinical follow-up was scheduled at two weeks, six weeks three months, six months, and 12 months then annually.

## Data analysis

Descriptive statistics were used to summarize the patients’ demographic, comorbidities, and radiological measurements. An unpaired Student *t* test was used to compare the quantitative data (Oxford hip score and SF12). The Mann–Whitney U test was used to compare pre- and post-treatment VAS scores. Frequency (percentage) and mean ± SD or median and range were used as appropriate for categorical and continuous values. The result was considered statistically significant if P value ≤ 0.05. All statistical analyses were done using statistical packages SPSS 23.0 (SPSS Inc. Chicago, IL) and Epi InfoTM 2000 (Centers for Disease Control and Prevention, Atlanta, GA).

## Results

In the study period, 15 patients (17 hips) underwent HBO for hip AVN; the mean age was 36 years, 47% were females, and 27% reported a known risk factor for AVN. Patients demographics are summarized in Table 1.

**Table 1:** Patients’ demographics

Total AVN number	15 patients (17 hips)	
Laterality	Unilateral	13
	Bilateral	2
Side	Right	9
	Left	8
Age (years)	36	
BMI	27.8	
Gender	Male	8
	Female	7
Number of sessions	39.5 ± 13	
	DM	1
	Smoking	4
	SCD	2
	Steroid	4
	No risk factors	4
Follow-up (months)	22.3	

*AVN* avascular Necrosis, *BMI* body mass index

Of the included patients, 73.3% were Steinberg stage II; 13 (86.7%) patients had satisfactory outcome with average final follow-up OHS score of 37.3 (Table 2). Four cases (26.7%) had stage progression on follow-up radiographs at final follow-up; three patients progressed from stage II to stage III and one patient from stage II to stage IV. Pain scores were collected from 13 patients at the final follow-up. The mean pre-treatment VAS score was 5.1 ± 2.1 compared to 1.5 ± 1.6 at the final follow-up (P < 0.001).

**Table 2:** Clinical functional outcomes

Outcomes	HBO		
	Satisfactory	Non-satisfactory	P value
Number	13 (86.7%)	2 (13.3%)	.001
OHS	37.3 ± 5.1	23 ± 5.6	.003
SF12 PCS	46.6 ± 9.5	36.9 ± 5.3	.192
SF12 MCS	44.5 ± 15.2	46.4 ± 5.2	.874

*OHS* Oxford hip score, *SF-12 PCS* 12-item short form survey physical component, *SF-12 MCS* 12-item short form survey mental component

There were no reported complications of HBO and none of the participants underwent any further surgical intervention in the form of core decompression or THA.

## Discussion

This study reported the outcome of a series of pre-collapse AVN of the femoral head in 15 consecutive patients treated with HBO. A significantly higher ratio of patients reported satisfactory outcome at the final follow-up (P = 0.001). Although progression of lesions to collapse was reported in 26.7% of cases, all of the progressed cases had a satisfactory outcome at the final follow-up with no further surgical treatment needed.

There are a small number of articles reporting clinical outcomes after HBO for femoral head necrosis, most of which were case series of limited cohorts of patients, and mostly with no control group nor comparison to other treatment modalities. A higher level studies in the form of prospective cohorts or randomized controlled trials are needed to more validate the future use of HBO in AVN of the femoral head.

## Clinical outcome

Camporesi et al. [8] randomized 19 patients with Ficat II femoral head necrosis to either HBO or hyperbaric air (HBA). The patients were exposed to 60 minutes of either HBO or HBA for a total of 30 sessions over a six week period. Both patients and outcome measure assessors were blinded to the treatment. The authors reported a significant improvement of pain scores in the HBO group after 20 and 30 treatment sessions with P = 0.002 and P < 0.01 respectively. The HBO group gained more degrees in all hip ROM after ten sessions; the increase was statistically significant (P < 0.001) except for hip flexion that was doubled in the HBO group but failed to show statistical significance. No side effects were reported in either group.

In the largest series of patients treated with HBO for AVN of the femoral head, Koren et al. [9] reported clinical outcomes of 54 patients (58 hip joints) with Steinberg stage I and II idiopathic, post-traumatic, and secondary AVN. All patients received six 90-minute HBO sessions per week with an average of 80 sessions per patient. Four joints (7%) had undergone hip arthroplasty at 11 years mean follow-up; all the four cases were secondary AVN. This concluded a 100% survival for idiopathic and post-traumatic cases.

Excluding the cases that underwent arthroplasty, the authors reported significant improvement of Harris hip scores (P < 0.0001) and the mental component of the short form 12 (SF-12) health survey (P < 0.0001). On further analysis, the improvement of Harris hip scores was still significant controlling for Steinberg stage and the aetiology of AVN (P < 0.0001).

In a prospective series by Vezzani et al. [10], all patients (8/8) with FICAT I and II AVN of the femoral head had post-treatment improvement of VAS pain scores in comparison to 27% (3/11) in FICAT III patients. The cohort received two cycles of 30 HBO sessions over three months. This was comparable with this cohort, where 12/13 patients (92%) had improvement of VAS score at the final follow-up, with only one patient reporting no change in pain score post-treatment.

A systematic review and meta-analysis on comparative studies was reported by Li et al. [11] in 2016; they included nine studies (2 in English and 7 in Chinese) with a total of 315 patients in the HBO group. They reported a 4.95 times higher clinical efficiency in the HBO group compared to the control group with statistical significance (OR = 4.95, 95% CI [3.24, 7.55], P < 0.00001). On subgroup analysis (Asian and non-Asian population), the clinical efficiency was still higher in the HBO group with 4.77 times higher in the Asian group (OR = 4.77, 95% CI [3.06, 7.44], P < 0.00001) and 7.07 times higher in the non-Asian group (OR = 7.07, 95% CI [1.77, 28.27], P < 0.00001).

## Radiological outcome

After the 30 sessions in the Camporesi et al. RCT [8], all HBA patients were offered six weeks of HBO therapy (cross over). Afterwards, all 19 patients underwent 90 HBO sessions over 12 months with an MRI offered at the end of the treatment. At seven years’ follow-up, the authors reported stable disease in all patients with no arthroplasty surgery needed with continuing radiographic improvement on the seven year MRI in 77% of patients compared to the 12 months MRI. Two patients demonstrated significant bone defect but with no clinical significance.

The same group prospectively followed a series of 19 patients with FICAT I–III post-traumatic and post-steroid AVN of the femoral head. The patients received two cycles of 30 HBO sessions/month with a no treatment break month in between (total of 60 cycles over 3 months). All FICAT I and II patients returned to normal MRI readings at one year follow-up. In contrary, 18% of FICAT III cases showed improvement on post-treatment MRI, 27% showed progression of collapse, and 55% showed stable lesions. The authors concluded that HBO is most effective when performed in earlier stages [10].

Reis et al. [12] compared the efficacy of HBO in 12 patients with Steinberg stage I to a cohort of untreated patient reported earlier by Vande Berg et al. [13]; an MRI inclusion criterion of subchondral lesions of 4 mm or more thick and/or 12.5 mm or more long was used in both studies. The HBO group received 100 sessions (6 days/week) and a follow-up MRI was done every three months for the first year and every six months in the second year. In nine patients (13 femoral heads) treated with HBO, the MRI was restored normal; two patients progressed to stage II AVN, the first one had systemic lupus and was on continuous steroids, and the other patient had idiopathic AVN. The last patient developed femoral head collapse and underwent total hip arthroplasty. The patient had chronic kidney disease and was on steroid treatment.

The authors further compared their results with the untreated group of the same lesion characteristics. They reported significantly lower likelihood of irreversibility of the lesion size in the HBO group (25%) compared to the untreated group (85%, P < 0.0001).

The same group reported the post-treatment MRI finding of 74 joints (64 patients) with Steinberg I and II AVN of the femoral head; the MRI was done two months after finishing an average of 80 sessions of HBO. Sixty-five out of the 74 joints (88%) showed reduction of the lesion size on post-treatment MRI. On sub-analysis, the percentage of improvement was higher in idiopathic AVN (93%) compared to post-traumatic (85%) and secondary AVN (75%). Furthermore, stage I cases showed 95% improvement in comparison to 81% in stage II cases [9].

Recently, Shier et al. [14] reported two cases of pre-collapse femoral head necrosis in patients with sickle cell disease (SCD). The first case was nontransfusion-dependent SCD and received twenty-eight 120-minute HBO sessions. The MRI at 6 months showed improvement from a lesion involving 45% of the femoral head (stage IC, severe) to involving 25% of the femoral head (stage IB, moderate). The second case was transfusion-dependent SCD and received thirty-nine 120-minute HBO sessions. Deterioration of the lesion from 35% of the femoral head with no definite collapse (stage IC, severe) to 45% of the femoral head with subchondral cystic changes (stage IIC, severe) was reported on one year MRI.

## Molecular biology

The receptor activator of NF-kB Ligand (RANKL), and the receptor activator of NF-kB (RANK) pathway is integral in bone metabolism. Overactivation of the RANKL/RANK pathway will shift the bone remodeling towards bone resorption. Osteoprotegerin (OPG) is a decoy receptor for RANKL, preventing RANKL binding to RANK, thus inhibiting bone resorption.

Vezzani et al. [10] studied the effect of HBO on the RANKL/RANL/OPG system in patients with AVN of the femoral head. They reported a significant increase in soluble OPG with the peak increment after 30 sessions of HBO (from 5.61 ± 1.99 pmol/L to 8.97 ± 2.07 [P < 0.01]). No significant changes were reported in RANKL levels. The authors concluded that HBO efficiency in AVN treatment could be explained by influencing the OPG/RANKL plasma levels.

Recently, Moghamis et al. [15] compared the clinical and radiographic outcomes in patients with Steinberg II AVN of the femoral head treated with either HBO or core decompression. Twelve hips were treated with core decompression, while 11 hips received a range of 25–40 HBO sessions. No significance differences in OHS or SF12 were reported with a P value of 0.13 and 0.67 respectively. 37.5% of the HBO group had AVN progression on one year radiographs compared to 62.5% in the core decompression group; however, no statistical significance was reported [P = 0.47].

In conclusion, HBO is considered a safe and effective treatment modality in pre-collapse early stages of AVN of the femoral head with low rates of lesion progression to collapse. This needs further validation by a higher level prospective large-scale trials.

## Data Availability

All data and materials will be furnished upon request.
